# Evolution and sustainability: gathering the strands for an Anthropocene synthesis

**DOI:** 10.1098/rstb.2022.0251

**Published:** 2024-01-01

**Authors:** Peter Søgaard Jørgensen, Vanessa P. Weinberger, Timothy M. Waring

**Affiliations:** ^1^ Stockholm Resilience Centre, Stockholm University, Stockholm, Stockholm 10691, Sweden; ^2^ Global Economic Dynamics and the Biosphere, Royal Swedish Academy of Sciences, Stockholm, Stockholm 10405, Sweden; ^3^ Anthropocene Laboratory, Royal Swedish Academy of Sciences, Stockholm, Stockholm 10405, Sweden; ^4^ Center for Resilience, Adaptation and Mitigation (CReAM), Universidad Mayor, Temuco, 4801043, Chile; ^5^ Mitchell Center for Sustainability Solutions, University of Maine Orono, ME 04473, USA; ^6^ School of Economics, University of Maine Orono, ME 04473, USA

**Keywords:** evolution, sustainability, Anthropocene, synthesis, evolvability

## Abstract

How did human societies evolve to become a major force of global change? What dynamics can lead societies on a trajectory of global sustainability? The astonishing growth in human population, economic activity and environmental impact has brought these questions to the fore. This theme issue pulls together a variety of traditions that seek to address these questions using different theories and methods. In this Introduction, we review and organize the major strands of work on how the Anthropocene evolved, how evolutionary dynamics are influencing sustainability efforts today, and what principles, strategies and capacities will be important to guide us towards global sustainability in the future. We present a set of synthetic insights and highlight frontiers for future research efforts which could contribute to a consolidated synthesis.

This article is part of the theme issue ‘Evolution and sustainability: gathering the strands for an Anthropocene synthesis’.

## 1. Introduction

Sustainability is about how humans can preserve opportunities for future generations on the planet. Evolutionary thinking about such human–environment interactions has a long history. However, while this thinking has continued to develop [1,2,18,19], (table 1) the sustainability challenge has become increasingly global and humans have become increasingly aware of its role in driving global change. Globally connected industrialized societies are now the main driver of change in the Earth system, a time period that has come to be known as the Anthropocene [20–24]. On the one hand, this ecological dominance of humans could be seen as the ultimate evolutionary success of a species. On the other hand, the overlapping systemic and global risks that mark the Anthropocene hardly seem like a success. Human activities are threatening the stability of the Earth's climate, the survival of many co-inhabitants, and indeed the basis for the stability of human civilization [21,25–27]. Dealing with these global sustainability challenges is the central question for whether human success will be long-lasting or short-lived, and whether it will be inclusive or for the few [24].

**Table 1.** Current frameworks for evolutionary thinking about human–environment interactions.

framework	description	key references
niche construction	theory describing how organisms change the environment and affect selection in the future	[1–5]
eco-evolutionary dynamics	framework of how evolution changes ecosystem-level processes and vice versa	[6–11]
social–ecological coevolution	systems perspective of feedbacks between human and environmental systems	[10,12–17]

Global sustainability in the Anthropocene has been scarcely explored from an evolutionary standpoint [1,28] and brings to the fore many challenging or uncharted topics for evolutionary studies. The fast pace of the Anthropocene encourages the use of diverse methods to study contemporarily unfolding dynamics. Its global nature and that we are all embedded in the system, necessitates multiple perspectives and begs the question of how evolution acts at the level of the evolving biosphere [29–33]. Finally, the global societal relevance and urgency emphasizes the need for transdisciplinary approaches [34,35].

**Figure 1.**
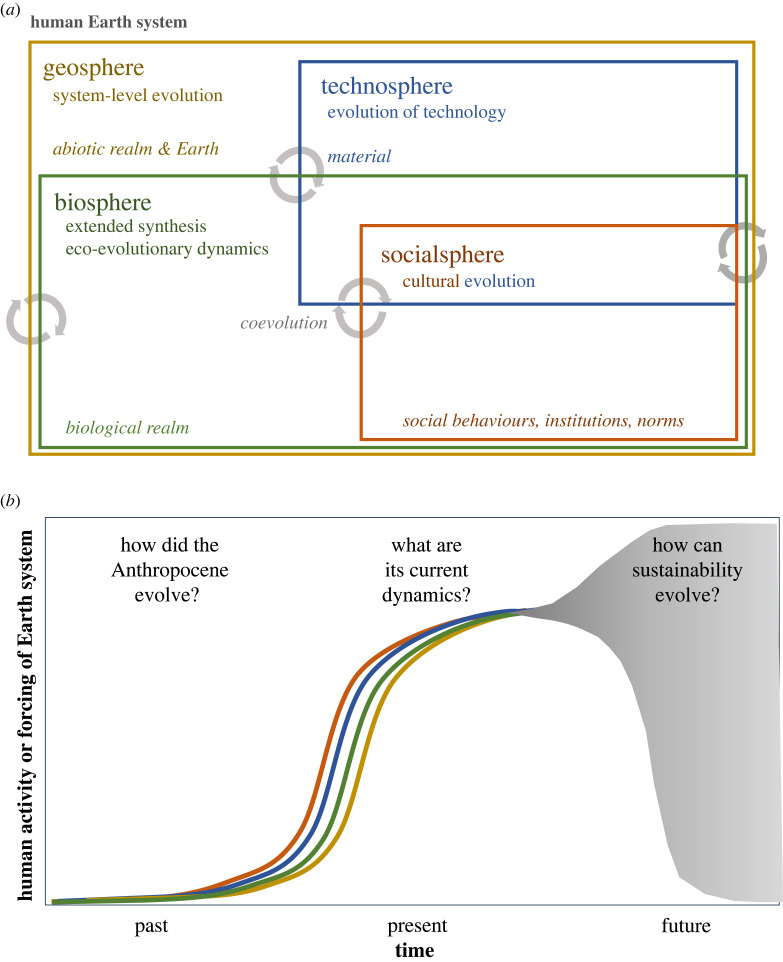
Evolution and sustainability in the human Earth system. (*a*) Evolution in the Anthropocene takes place within and between (arrows) four overlapping and embedded spheres. Some of the major theoretical frameworks are indicated for each sphere along with some of the phenomena they pertain to (italics). (*b*) The three-time horizons of the Anthropocene synthesis and the major question asked in each section (Online version in colour.)

Given the diversity of perspectives needed for understanding the multiple timescales and levels at which evolutionary dynamics unfold in the Anthropocene, it is useful to start with an inclusive definition of evolution. Evolution is essentially about the dynamics of innovation (generation of variation) and differential transmission (selection and inheritance). These dynamics can take place within and across multiple embedded, overlapping and coevolving domains of the Earth system (figure 1*a*). These domains include the non-living parts of Earth (the geosphere), the biological realm (the biosphere), human socially transmitted and constructed information (what we might call the socialsphere), and constructed material technologies, living or not (the technosphere). The latter two domains are sometimes referred to simply as the anthroposphere [1,36–43].

There are indications that both evolutionary and sustainability sciences are ready to engage in an Anthropocene synthesis. In the evolutionary sciences, there has been a recent proliferation of integration and synthesis of increasingly interdisciplinary character, relevant for all domains of the human Earth system. These include evolution of non-living matter and system level evolution [44], eco-evolutionary dynamics [6,45,46], cultural evolution and extensions to environmental social science and policy [1,47–55], the evolution of technology and the technosphere [56–58] and the extended evolutionary synthesis [3,59–61]. Meanwhile, sustainability science, a problem-oriented and interdisciplinary field spanning the sciences, humanities and practice, has grown tremendously in the past decades [24,62,63], gone through its own processes of synthesis, borrowed and further developed many evolutionary metaphors and analogies [e.g. 64–66].

At present, there are many individual strands that need gathering for an Anthropocene synthesis of evolution and sustainability. Evolutionary dynamics of human-environment interactions are commonly studied through frameworks, such as social-ecological coevolution, niche construction and eco-evolutionary dynamics, but with little interaction between these approaches (table 1). As we also see increasing application of these perspectives to understand the Anthropocene and global sustainability [1,18,29,34], there is clearly a need for consolidating our own symbolic representation of how humans evolve with the environment.

This article introduces a theme issue which explores and advances the state of the art of an Anthropocene synthesis. The issue presents a diversity of topics and methods of inquiry that should be considered in that effort. It offers the first glimpse of what an Anthropocene synthesis might look like, covering, methods, theories, empirical approaches and time frames. We have divided these into three sections corresponding to the three time horizons we need to understand: the past, present and future of the Anthropocene (figure 1*b*). Here, we review this body of research, highlight insights and common themes, evaluate the overall status of an Anthropocene synthesis and sketch the outline of a larger research agenda.

## 2. Past: how did the Anthropocene evolve?

**Figure 2.**
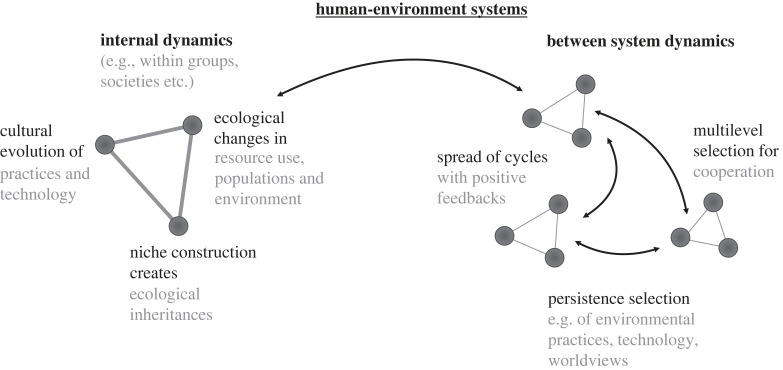
Major processes involved in the evolution of the Anthropocene and their interactions in dynamics within and between human-environment systems.

The first major task in developing an Anthropocene synthesis is to understand humans' increasing influence on the global environment as it emerged from human evolution. How the Anthropocene evolved is connected to the study of the unique evolution of the human species. Important patterns include the explosive population growth of *Homo sapiens sapiens*, the expansion of human use of resources, the increased human social and technological complexity, and associated increases in scale of organization. The articles in this section explain the evolution of the Anthropocene with various traditions in evolutionary theory, including human cultural evolution, niche construction, the evolution of complex systems and evolutionary transitions (figure 2).

### (a) The cultural transition and human agency

It is impossible to understand the foundations for the Anthropocene without first understanding that humans are a species with the ecological advantage of a system of cultural evolution that accumulates over time. The human transition to becoming a cultural species has been the subject of immense amount of work [67–74]. While the emphasis on details differs, there is agreement that language is key to securing fidelity in the transmission of social information and that cognitive capacity for symbolic language and representation as well as enhanced mental flexibility for domain-general thinking are foundational [67]. Another way to summarize the transition to culture is through the lens of increasing human agency, the capacity to determine and take conscious action to pursue a goal [75,76].

In their contribution to this issue, Richerson *et al*. [77] provide a refreshing perspective on human agentic forces that they in their landmark contribution from 1985 set apart from the blind forces of cultural evolution [78]. The authors distinguish the blind forces of cultural evolution, such as cultural drift and natural selection on culture from the agentic forces of cultural evolution, such as learning and creativity. They argue that human agency drove the evolution of the Anthropocene through the development of short-term resource exploitation behaviours, which facilitated a runaway process of cumulative culture evolution. They then propose that agentic forces are already in use, but need to be more actively applied to address today's global sustainability challenges with policy that focuses on long-term outcomes for paving a more sustainable human trajectory. Re-emphasizing the central role of agency in cultural evolution is important as it is an important concept in the social sciences, and removes any misunderstanding that evolutionary perspectives are only about blind forces. More work is needed to explore how human agency can shape cultural evolutionary processes and outcomes at the global level, which we return to in §4.

### (b) Multi-level selection and increasing levels of cooperation

One of the strongest trends that characterizes the Anthropocene is the increase in the scale of human social organization, culminating in a globally connected social–ecological system [1]. The emergence of higher levels of organization and cooperation is sometimes the result of evolutionary transitions in individuality [79]. Such transitions have been important in the evolutionary history of the planet and help to explain the emergence of the first eukaryotic and multicellular organisms [80–83]. The evolutionary transitions theory may also help explain the emergence of human culture and society [84]. Whereas the transition to humans as a cultural species is often studied through the lens of gene-culture coevolution, the transition to humans as an increasingly socially cooperative species is studied through the framework of multi-level selection. Recent research proposes that human evolution can be described as a partial or ongoing evolutionary transition involving both inheritance and individuality [67,85,86]. In this issue, Waring *et al*. [87] suggest that the process of cultural group selection is the central evolutionary process that drove the Anthropocene and human domination of the biosphere (see §4).

### (c) Positive feedbacks in human niche construction

A fundamental human capacity involved in creating the Anthropocene is the ability of our species to shape its own environment, including through domestication, production ecosystems, settlements and technological infrastructure. The transition to becoming a cumulative cultural species has allowed humans to shape the environment in a complex manner. Such impacts, their feedbacks and the evolutionary implications are the topic of niche construction theory [5], which can be extended to cultural species through the lens of cultural niche construction [88,89]. Niche construction involves both positive (growth opportunities) and negative (limiting) feedbacks on human societies. The social and environmental feedbacks that control human population involve the accumulation of technology, social structure and the benefits of cooperation in larger groups. The link between niche construction and cooperation at increasing social scales has been linked through the concept of sociocultural niche construction [1]. This theme has a long history of study from Malthus [90,91] to Boserup [92] and beyond. Broadly, we can consider three types of feedback involved in human niche construction: technology and environmental modification (i.e. tools, infrastructure), social scale (i.e. population size, connectivity) and social capacity (i.e. cooperation, knowledge, power). These factors may also positively reinforce each other as well, leading to qualitatively different population dynamics for humans relative to other niche-constructing species.

Three contributions in this issue further explore the role of these positive feedbacks in creating the Anthropocene. Efferson *et al*. [93] explore the behaviour of a dynamical model with two types of technologies subjected to cultural evolution: one that expands the regular carrying capacity of resources relevant to humans (production technology) and one that optimizes the efficiency of their accessibility for human consumption (consumption technology). They find that the model produces a wide variety of dynamics and exhibits extreme sensitivity to initial conditions (path dependency), and they observe cycles of super-exponential growth and collapse. Super-exponential growth patterns also emerge in the empirical investigation of Lima *et al*. [94]. Comparing demographic patterns of prehistoric populations in regions around the world, they find reproduction curves and population signatures that match the more recent population explosion of many industrialized countries. The authors characterize this effect as resulting from the positive feedback between evolving production technology and population size, paralleling the modelled dynamics of Efferson *et al.* [93]. The congruence between these theoretical and empirical findings are notable. It hints that human population dynamics may be unique and should be further investigated in light of the interaction between cumulative cultural and technological evolution, cooperative production systems and environmental feedbacks from e.g. natural resources stocks.

Gayo *et al*. [95] investigate a particularly interesting example of population growth and collapse, that of the Atacama Desert and the agricultural inhabitants that lived there for the last 2000 years. Their findings support the view that the human–environment feedback loop appears closely linked with patterns of cooperation or competition between individuals, modulated by both environmental and social factors. The authors conclude that warfare and droughts increased competition among individuals, associated with bust dynamics; while social upscaling and peace periods (or lack of conflicts) enhanced their cooperation, leading to booming population growth. This finding suggests that the regulation of cooperation in human groups may have been a major evolutionary force of change underlying the Anthropocene. All three papers suggest that culture, technology, cooperation, and social scale and structure play a role in the environmental ratchet that created the Anthropocene.

### (d) The spread of systems

Evolutionary processes can also apply to systems, causing them to gain certain patterns or structures over time. A recent advance in this domain has been the formalization of system-level means of selection through the mechanisms of selection by persistence and differential spread [29–32,44]. In persistence selection, a generative environment, such as the Earth's biosphere, creates alternative configurations over time. Unstable configurations collapse, followed by the emergence of a new configuration which may have different stability properties. In this way, persistence selection is a type of evolutionary change in a population of one single complex, reconfigurable system.

Persistence selection was developed to try and explain how a stable biosphere could evolve. Persistence selection is therefore relevant for understanding the stable Holocene environment that, as many scholars have suggested, allowed humans to use their cultural capacities to build complex societies [96]. Recently, it has been proposed that this mechanism may also be useful to understand the crisis of unsustainability in the Anthropocene [29]. Two papers draw on the importance of persistence selection and differential spread as an explanation for Anthropocene evolution. Both papers suggest that social–ecological system structure is a critical feature for explaining the origins of the Anthropocene at both the level of individual societies, cities or regions and at the level of the entire planet.

Lenton & Scheffer [97] argue that the differential spread of human systems with certain reinforcing feedbacks have been a major mechanism in the origins of the Anthropocene. They argue that feedback processes enabled the spread of human systems, creating a general type of selection mechanism acting on ecosystems. The authors suggest that system traits which have facilitated the Anthropocene trajectory include fire use, agricultural development, state formation, labour specialization and industrial investments. Like Richerson *et al*. [77], Lenton & Scheffer [97] conclude that such positive feedback loops will have to be purposefully engineered to prioritize persistence to deal with the Anthropocene sustainability crisis.

Weinberger *et al*. [98] take an additional step by developing a toy model to study how such human systems might spread and work to characterize their dynamics. The authors model the evolution of ecological systems using energy flow networks. They compare greedy scenarios (representing the growth of human social systems) in which a strategy with high energy consumption (e.g. on certain nodes or energy forms) drives overall system change to scenarios without such greedy strategies. In general, greedy strategies compromised system stability characteristics across a range of metrics, including scarcity tolerance. However, greedy networks that achieve greater total power also exhibit better scarcity tolerance, but those configurations seldom evolved. Weinberger's *et al*. [98] model highlights the possibility of formally combining questions of system-level evolution with population-level evolution. Such research represents a new theoretical frontier in evolutionary sustainability science. Further study is needed to understand the conditions under which global system-level persistence selection might predominate, thereby possibly leading towards sustainable equilibrium conditions.

## 3. Present: evolutionary dynamics of the Anthropocene

**Figure 3.**
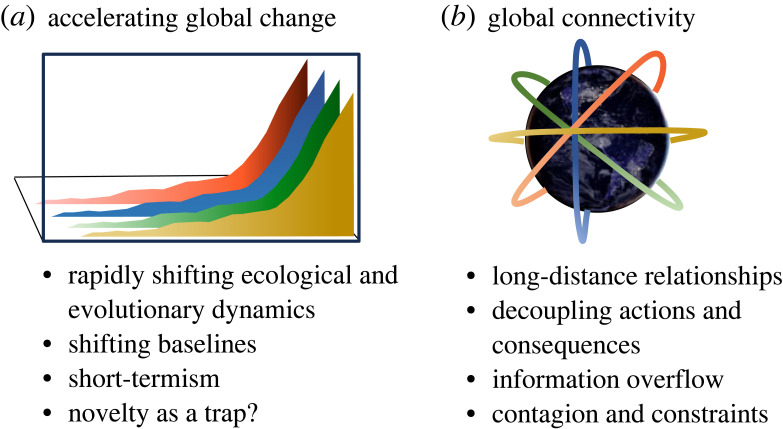
Factors shaping current Anthropocene dynamics in the human Earth system. (*a*) Accelerating global change and associated dynamics. (*b*) Global connectivity and associated dynamics.

The Anthropocene has its own set of complex dynamics that must be navigated to set humanity on a path towards sustainability. The Anthropocene system is characterized by rapid change, high levels of global connectivity, resulting in new forms of experienced inequality [99,100] (figure 3). These three forces, together with the seemingly ever-increasing power of new technologies, combine to shape present day global sustainability challenges [24,101,102]. This second section focuses on the medium timescale and the current evolutionary dynamics of sustainability challenges. The section consists of six investigations on topics such as evolutionary traps, short-termism, long-distance relationships, niche construction in global systems, eco-evolutionary dynamics in urban areas, and the evolution of a global consciousness. Together, the contributions reveal a set of interacting dynamics underlying interconnected global risks and challenges [103–105].

### (a) Accelerating change and shifting baselines

The Great Acceleration describes the growth in the scale of human societies and changes to the environment during the twentieth century [100] (figure 3*a*). In less than a century, the human population increased *fourfold*, the economic output *sixfold*. Along with these changes have been widespread global environmental and social change. The pace of change in the Anthropocene poses new challenges, including shifting baselines and the need to manage rapid cultural and biological evolution in the biosphere.

From a perspective of cultural evolution, where social transmission is a foundational process, accurately transmitting historical baselines becomes difficult during times of rapid global change. This leads to the risk of shifting baselines, which can reinforce behaviours acting on short-term historical and future information. The degree to which humans today act to increase wealth and consumption as if there were multiple planets to exploit shows the global ecological consequences of shifting baselines.

Shifting baselines have been well established as an ecological phenomenon in sustainability science [106–108] but have yet to be formalized as a cultural evolutionary phenomenon. Kemp *et al*. [109] summarize insights from innovation and socio-technical transitions studies and emphasize how actors often end up being caught by optimizing for shorter-term or rapidly unfolding evolutionary processes at the expense of longer-term goals, like sustainability. They highlight how short-term goals tend to drive policy and action in the Anthropocene.

Eco-evolutionary dynamics focuses on the interplay between ecological and evolutionary change [6,9,11]. The rapid growth of intensively managed human ecosystems, such as settlements and production systems, causes rapid changes in the composition of the biosphere. Some species rapidly adapt to these human environments, often in ways that challenge human governance strategies [34,110,111]. Alberti [112] looks at the interrelated evolutionary, ecological and sociocultural dynamics in cities. Cities are not only major drivers of global social and environmental change and home to more than half the human population, but also hotspots of rapid biological evolution. Alberti shows how species inhabiting cities are undergoing contemporary evolutionary changes and how these can be incorporated into the design of urban green and blue infrastructure to enhance the sustainability of local management regimes. At the same time, cities are a hotspot for global human sociocultural evolution. Being able to manage local eco-evolutionary dynamics could help accelerate broader action for sustainability beyond the city limits and as such help change the Anthropocene current trajectory [113–115].

### (b) When everything is connected

The highly connected global systems of the Anthropocene mean that distant actors and actions are interrelated in concrete and diffuse ways, almost so that any action can affect everyone and anyone can affect everything [116–118]. In sustainability science, there are multiple frameworks for investigating these dynamics, including cross-scale dynamics, teleconnections and telecoupling frameworks [118–122]. In evolution, there have been fewer attempts at understanding these dynamics beyond those of information transmission, e.g. using digital technology [123,124].

In this issue, multiple contributions start unpacking the evolutionary dynamics of global connectivity. Using empirical case studies from Africa, Pisor *et al*. [125] look at the multiple roles long-distance social relationships can have on local natural resource management and conservation efforts. They conclude that such relationships can either undermine or facilitate sustainable management as they can either transmit norms of overharvesting or sustainability. Dorninger *et al*. [126] explore the effects of niche construction in globally connected Anthropocene systems. The authors argue that in the Anthropocene, niche construction can have selective effects on distant social–ecological systems, which can promote unsustainable behaviours as exposure to the costs of these behaviours are physically removed. Thus, high levels of connectivity can incentivize unsustainable behaviours. The study and management of connectivity and its effects are therefore important.

One important building block for addressing the challenges brought about by global connectivity is a shared awareness or global perspective that shapes behaviour. How might such a ‘global consciousness' evolve? Zhang *et al*. [127] explore this possibility with an empirical study of correlates of a psychological construct which is associated with both pro-environmental behaviour and identification with all humanity, termed ‘global consciousness’ (GC). The authors report results from a cross-cultural study finding that GC increases with education and religion, decreases with age and that various life events, such as promotion, death and marriage, all can help increase expressions of global awareness in the short term. Future research can explore the factors that allow GC to expand or cause it to contract.

### (c) Polycrisis: the Anthropocene as an evolutionary trap

The complex architecture of the Anthropocene may be beyond our capacity to navigate for desirable outcomes. Just like human-induced novelties often serve as evolutionary traps for other species [128–131], the Anthropocene could also be a large-scale evolutionary trap for our own species. Søgaard Jørgensen *et al*. [102] ask whether the Anthropocene may be as much an evolutionary trap for humans as human-driven global change is for many other species, such as seabirds eating marine plastics or the many islands faunas naive to introduced predators. They identify 14 types of evolutionary traps and find that these traps often reinforce each other in an Anthropocene polycrisis. For many traps, there is evidence of increasingly hard-to-reverse dynamics and emerging signs and risks of declining well-being.

## 4. Future: Anthropocene transitions, evolvability and science for change

**Figure 4.**
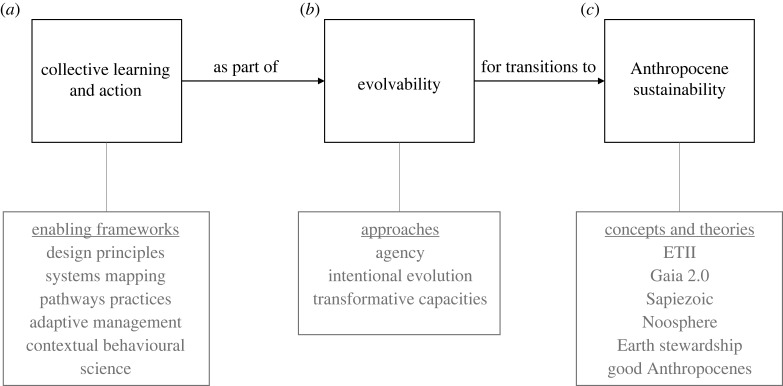
The relationship between theories concerned with and key capacities needed for the future evolution of global sustainability in the Anthropocene. Collective learning and action is an important component of evolvability for Anthropocene sustainability. Examples of frameworks, approaches, concepts and theories are listed.

Given the systemic and interrelated challenges of the Anthropocene, it is likely that large-scale changes are necessary to move towards global sustainability [24,132–134]. However, how do we conceptualize global sustainability in relation to the Anthropocene? What are the key processes and principles involved in such a transition to sustainability? Also, how can knowledge best be generated to advance the interdisciplinary interface between evolution and sustainability? In the third section of the theme issue, six contributions provide insights relevant to these questions (figure 4).

### (a) Global sustainability and the ‘one planet problem’

Tthe human species has been conceptualized as a global force of change even before the seminal early works of Vernadsky and Teilhard de Chardin on the Noosphere [22,135,136], and much longer before our interaction with recent new forms of technology [127,137,138]. Several conceptions of global sustainability clearly indicate a transition in how humans behave as a cultural species at the planetary scale (figure 4). This is the case with concepts such as Gaia 2.0 [139,140], Earth stewardship [99,141] and perhaps most explicitly, the Sapiezoic [142]—a hypothetical new aeon where humans are conscious stewards preserving a stable Earth system. Other conceptions of Anthropocene transitions, such as the seeds of the good Anthropocene [134], emphasize the importance of enabling and scaling bottom-up change. Multiple contributions in the theme issue elaborate these transitions and mechanisms.

Waring *et al*. [87] suggest that the Anthropocene was caused by a characteristic pattern of cultural group selection acting on collective patterns of human environmental use [85]. They argue that global traits for environmental management are needed to achieve global sustainability. However, this poses a problem. Since mechanisms selecting for cooperation at the global level are weak compared with lower levels of the hierarchy, the necessary traits and systems for global environmental management cannot evolve. As such, the authors suggest that current modes of human cultural evolution are at odds with the possibility of a single sustainable planet. This ‘one planet problem’ presents an important and controversial issue for further scrutiny. Future research should seek to answer how a transition to global sustainability might evolve and what mechanisms would be involved. The ‘one planet problem’ is a profound challenge for Anthropocene sustainability, at least in theory as cultural multi-level selection has been central to evolutionary thinking on cooperation, including for sustainability [19,80,81,83,143].

One way to advance transitions to sustainability without the need for global cooperation might be by amplifying positive messaging about the capacities of humans to deal with complex problems. Ellis [144] describes the Anthropocene as a condition of coevolving social–ecological entanglement in which transformative changes are ongoing, inevitable, and differentially caused and experienced by different social groups. He argues that many historic and prehistoric societies adapted to, transformed themselves, and thrived through unprecedented social–ecological challenges, and that contemporary societies have even greater sociocultural capabilities to address the planetary environmental challenges of the Anthropocene. The question for current sustainability challenges, he argues, is not whether societies can shape a better future for people and planet, but whether the capabilities and responsibilities to accomplish these better futures will be recognized and demanded broadly enough to achieve the larger scales of societal agency needed to achieve them.

### (b) Inequity inhibits the evolution of collective action

Inequality and injustice, whether material or perceived, are one of the main characteristics that can significantly hamper cooperation for global sustainability [145]. A pertinent example here is the inequality in historical greenhouse gas emissions and perceptions of just burden sharing to mitigate climate change [146]. Safarzynska & Smaldino [147, p. 1] show with a simple model how the presence of a global externality beyond the local group level and the distribution of impacts from this externality can affect the cultural evolution of local prosocial behaviours. They find that ‘the global externality promotes the evolution of local cooperation only if it either disproportionately benefits the poor or disproportionately reduces the payoffs of the rich. If the global externality primarily harms the poor, it undermines the evolution of local prosocial behaviour’.

Access to information is another important form of inequality in the Anthropocene. Transparency initiatives seek to counter such inequality by increasing information available for consumers, e.g. with pro-environmental preferences in global supply chains [148]. Demonstrating the importance of transparency, Von Flüe *et al*. [149] provide a model of green consumer preferences and how they, combined with asymmetric information access between businesses and consumers, can help sustain greenwashing. While transparency might not be sufficient for sustainability, the authors argue that it is probably necessary as the lack of transparency can easily sustain unsustainable activities. This finding connects ideas in the economics literature on the impact of asymmetric information to cultural evolutionary research on sustainability, opening a door for new interdisciplinary work.

### (c) Evolvability

The science presented in this issue makes it clear that to approach sustainability in the Anthropocene societies will need to evolve. In biological organisms, the concept of genetic diversity, modularity and phenotypic plasticity are three important components of evolvability, the ability to evolve. However, for cumulatively cultural human societies, much less work has been done on operationalizing this concept. Two papers in the theme issue argue that more work is needed to understand capacities that will enable societies to evolve [102,144], including the capacity to design interventions that direct cultural evolution in collectively agreed directions [150].

The papers highlight that while little work has been done in the evolutionary sciences *sensu stricto*, a lot of work has been focusing on these questions in rapidly growing fields such as transition and transformation studies and studies of social–ecological resilience. In resilience studies, the capacities of actors to persist, adapt (evolving within the adaptive landscape) and transform systems (capacities to change the structure of the adaptive landscape) have been central concepts for more than a decade. Bringing concepts such as transformative capacities into a more explicit evolutionary framework is a major priority for the forward-looking part of evolutionary sustainability science [151]. Likewise, several papers emphasize the need for intentional evolution for finding solutions to the global sustainability challenge, which highlights the importance of renewing the focus of early work on agency [75,76,152,153] as part of studying evolvability of actors and systems at multiple levels. Here there is an interesting schism between the agentic forces that re-enforce business-as-usual and the form of agency to disrupt those forces.

### (d) Designing processes and environments that enable collective action

An important aspect of evolvability will be the ability to design settings that enable learning and collective action (i.e. cultural evolution) for global sustainability (figure 4). There are many frameworks that engage with evolutionary processes, many from other fields than evolution *sensu stricto* [151]. Examples include frameworks for experimentation and learning, such as through adaptive management [17,154], transition frameworks, like three-horizons that encourages actors to envision a sustainable future and map the necessary birth-and-death processes in a system of their interest (www.h3uni.org) [155]. A basic aspect of enabling collective action is to make groups work well. Here an interesting example is the ProSocial process (www.prosocial.world). ProSocial is unique in explicitly drawing on multi-level selection, intentional cultural evolution, Ostrom's design principles and acceptance and commitment therapy from psychology, which help individuals as well as groups pursue their goals [156,157]. Given the ‘one planet’ problem, such practices are particularly needed when they are applied to form new groups with potential to alter the dynamics of global sustainability on one or more sectors [158]. Examples here are still few, but include SeaBOS, a scientist-enabled coalition of some the largest seafood companies working to make the industry more sustainable (www.seabos.org). Initiatives such as SeaBOS illustrate how scientists can play an active role in facilitating the formation of such groups [159,160].

### (e) The interdisciplinary dialogue

Science is today an important sector of the global economy and can influence Anthropocene trajectories. The capacity of science itself to evolve is, therefore, also a relevant aspect of overall societal evolvability. One component of the evolvability of science for sustainability is the exchange, innovation and consolidation of approaches, theories and methods between disciplines with the aim of reducing silofication and pursuing major breakthroughs. Currie *et al*. [161] demonstrate and explore this dialogue between social–ecological systems and cultural evolution research. They highlight the basic steps that are often left out of interdisciplinary efforts, causing them to fail. These include bilingual glossaries which link terms with equivalent meaning, and more importantly, flag those terms which are equivalent in name but hold different meaning between fields. They provide novel examples of how traditions from the two fields can be integrated, e.g. in the form of systems mapping (a common practice in social–ecological systems) that centres on mapping of evolutionary processes.

## 5. The frontiers of an emerging synthesis

In the previous three sections, we have reviewed some of the major strands of an emerging Anthropocene synthesis of evolution and sustainability. In this section, we focus on some of the frontiers that subsequent work will have to address, spanning empirics, theory, methods, new concepts and translation to action.

### (a) Comparing theories of Anthropocene sustainability

One priority in the next phase of the Anthropocene synthesis will be harmonizing and formalizing various narrative theories of Anthropocene evolution and sustainability. Where possible, this can include translation to mathematical modelling frameworks. Mechanistic theories of how global sustainability could emerge in the Anthropocene are needed. Currently, there are multiple such theories, some optimistic and some more pessimistic. These theories should be formalized, compared, scrutinized and tested. This will require fundamental theoretical innovation.

### (b) Evolution in systems and populations

The interface between systems thinking and population thinking is an area that is stimulating major creativity and advances in thinking about the Anthropocene. Research papers in this issue use one framework or another. However, few, if any, models combine the evolution of populations with system evolution. We suspect this may be necessary, and understanding the relationship between the two is certainly important. Explicitly integrating multiple inheritance and transmission systems with multi-level dynamics in such frameworks will be an interesting avenue for modelling sustainability in an Anthropocene composed of interlinked and evolving systems-of-systems or what we might call complex evolving systems.

### (c) Strengthening empirics and models

One of the first priorities in the next phase of the Anthropocene synthesis will be strengthening empirical analysis. Much work on Anthropocene evolution and sustainability presented in this issue consists of relatively simple mathematical models. To enhance applicability, models must be formulated in a manner that is suitable for parametrization and analysis using data from the real world, such as nation-states, corporations and (pre-)historical societies. To facilitate such a transition, inventories of data for analysing human–environment evolution will be useful [108]. Such efforts could also enable evolutionary dynamics to be included in integrated assessment models and modelling of human–Earth system dynamics.

### (d) Innovation systems

The extended evolutionary synthesis has established the concept of multiple inheritance systems. Dorninger *et al*. [126] argue that the nature of cultural inheritance most relevant in driving human–environment interactions is often blended. When it comes to the sources of human innovation for sustainability, it is clear we need multiple systems to provide novelty in ecological, material and social domains. For example, biomimicry has been used to enhance technological innovation, using the biosphere as an explicit source of inspiration [162–166].

### (e) Evolvability

Capacities for undergoing evolutionary change are another area of recent attention that deserves more work. This includes the areas of intentional evolution and design and higher-level transformative capacities in societies. Next steps include going beyond conceptualization to measurement of such capacities.

### (f) From science to action

An aspect of evolvability in society itself will be the capacity of scientists to translate their insights into action. Here, co-production and co-learning with practitioners will be an important practice to adopt from sustainability science. This will allow evolutionary thinking to become an important player in the Anthropocene. Evolutionary scientists should be able to demonstrate how evolution adds value to the current state of action-oriented sustainability science in the Anthropocene.

## 6. Conclusion

There are many strands converging on the topic of evolution and sustainability in the Anthropocene in all its diversity. We hope this theme issue helps highlight the many opportunities for consolidation of these strands to speed up one of the most important and challenging issues for science in our time. Finally, we wish to thank all contributors to the issue, including those working behind the scenes with reviews and other guidance.

## Data Availability

This article has no additional data.
